# Inhibition of serum- and glucocorticoid-induced kinase 1 ameliorates hydrocephalus in preclinical models

**DOI:** 10.1186/s12987-023-00461-0

**Published:** 2023-08-18

**Authors:** Alexandra Hochstetler, Hillary Smith, Makenna Reed, Louise Hulme, Paul Territo, Amanda Bedwell, Scott Persohn, Nicola Perrotti, Lucia D’Antona, Francesca Musumeci, Silvia Schenone, Bonnie L. Blazer-Yost

**Affiliations:** 1 Department of Biology, SL358, Indiana University Purdue University Indianapolis, 723 West Michigan Street, Indianapolis, IN 46202 USA; 2 Department of Medicine, Indiana University School of Medicine, Indianapolis, USA; 3 Dipartimento di Scienze della Salute, Università” Magna Graecia” di Catanzaro, Catanzaro, Italy; 4 Department of Pharmacy, University of Genoa, Genoa, Italy

**Keywords:** Hydrocephalus, Choroid plexus, Transepithelial epithelial ion transport, Serum- and glucocorticoid-induced kinase 1

## Abstract

**Background:**

Hydrocephalus is a pathological accumulation of cerebrospinal fluid (CSF), leading to ventriculomegaly. Hydrocephalus may be primary or secondary to traumatic brain injury, infection, or intracranial hemorrhage. Regardless of cause, current treatment involves surgery to drain the excess CSF. Importantly, there are no long-term, effective pharmaceutical treatments and this represents a clinically unmet need. Many forms of hydrocephalus involve dysregulation in water and electrolyte homeostasis, making this an attractive, druggable target.

**Methods:**

In vitro, a combination of electrophysiological and fluid flux assays was used to elucidate secretory transepithelial electrolyte and fluid flux in a human cell culture model of the choroid plexus epithelium and to determine the involvement of serum-, glucocorticoid-induced kinase 1 (SGK1). In vivo*,* MRI studies were performed in a genetic rat model of hydrocephalus to determine effects of inhibition of SGK1 with a novel inhibitor, SI113.

**Results:**

In the cultured cell line, SI113 reduced secretory transepithelial electrolyte and fluid flux. In vivo, SI113 blocks the development of hydrocephalus with no effect on ventricular size of wild-type animals and no overt toxic effects. Mechanistically, the development of hydrocephalus in the rat model involves an increase in activated, phosphorylated SGK1 with no change in the total amount of SGK1. SI113 inhibits phosphorylation with no changes in total SGK1 levels in the choroid plexus epithelium.

**Conclusion:**

These data provide a strong preclinical basis for the use of SGK1 inhibitors in the treatment of hydrocephalus.

## Background

Cerebrospinal fluid (CSF) surrounds the brain, cushioning it and providing effective weight reduction. In addition, this unique fluid provides nutritional and hormonal support, is instrumental in removing waste products, and circulates signaling molecules and immune cells [1]. Interestingly, the amount and composition of the CSF is modulated on a diurnal basis and may be involved in the control of sleep/wake cycles, underscoring the importance of regulation of this fluid for normal physiological function [2].

In humans, CSF is produced and reabsorbed at a rate of approximately 500 mL/day [1]. This vital fluid flows throughout the brain ventricular system and central canal before being reabsorbed. Viewpoints on how this amount of CSF is reclaimed in the absence of a CNS parenchymal lymphatic system have evolved over the past several decades. In the old “textbook version” CSF absorption was envisioned as occurring via arachnoid villi or granulations [3]. More recently, there is some consensus in the literature that the lymphatics of both olfactory submucosa underlying the cribriform plate [4] and the more recently elucidated meningeal-dural sinus [5] play major roles in this process. Proulx has provided an excellent historical review of this subject [6]. The balance between production and absorption can become dysregulated in conditions such as traumatic brain injury, hydrocephalus, stroke, or poorly understood age-related disorders [7, 8]. In hydrocephalus, a disruption of CSF flow or a mismatch between production and reabsorption results in CSF accumulation that can damage brain structures. Left untreated, hydrocephalus can result in cognitive impairment, developmental delay, brain damage, gait instability, vision loss, sleep disturbances and death [8–10].

The current standard of care for hydrocephalus is to create a CSF drainage route, either by the placement of a physical device (shunt) or the creation of a new pathway (endoscopic third ventriculostomy) [8, 10–13]. Although shunt placement is the most common form of intervention, it is wrought with complications including infection, blockage and mechanical failure and approximately 50% of all shunts in children will require a revisional surgery within two years [11, 12]. Although the third ventricle endoscopic procedure is successful in a subgroup of patients, few qualify for this procedure due to the etiology of their hydrocephalus [13]. A durable and effective pharmaceutical treatment for hydrocephalus is, therefore, necessary and would provide a non-invasive, long-term option for patients with hydrocephalus.

The choroid plexus (CP), which produces the majority of the CSF, is composed of a fenestrated capillary network surrounded by a barrier epithelium, forming the blood-CSF barrier [1, 14, 15]. The choroid plexus epithelial cells (CPe) are responsible for the secretion and unique composition of the CSF via the regulation of multiple CPe transporters and channels [1, 15–18]. While the identity of the channels and transporters are well-characterized, the regulation of these entities is less well understood. Two of these channels, the non-specific cation channel, transient receptor potential vanilloid 4 (TRPV4) and the sodium, potassium, 2 chloride channel 1 (NKCC1), have recently been implicated in the pathophysiology of hydrocephalus [19–21]. Our group has previously shown that TRPV4 is expressed in both native choroid plexus and in two choroid plexus-derived continuous cell lines and is a major regulatory hub channel in the CPe [22–25]. TRPV4 responds to multiple stimuli including changes in osmolarity, temperature, mechanical stress, and inflammation [26, 27]. The current studies highlight a novel drug target, serum- and glucocorticoid-induced kinase 1 (SGK1) within the TRPV4 pathway and show the efficacy of an inhibitor of SGK1 in the treatment of hydrocephalus in vivo and mechanistically in a human cell line in vitro.

SGK1 was first identified in a mammary tumor cell line [28] and subsequently found to be activated by a range of stimuli including hormones, serum, and cell volume changes [29–31]. Early studies characterized SGK1 as a convergence point in the stimulation of the renal epithelial sodium channel by both steroid and peptide hormones [32]. Subsequent studies indicated this kinase to be an important signaling intermediate in the regulation of a number of channels and transporters across multiple tissues [31]. More recently SGK1 has been implicated in oncogenic transformation [33–37]. SGK1, as a pivotal kinase induced during cell stress [30, 31], has been allied with the stress-induced channel TRPV4 in the lung during ventilator-induced damage [38] and in cultured cells where TRPV4 is an SGK1 substrate [39] and phosphorylation by SGK1 is required for the binding of actin [40]. In transfected cell studies, phosphorylation by SGK1 was shown to promote single channel activity of the TRPV4, Ca^2+^ influx, protein stability and an expansion of the cell membrane [40]. It is likely this interaction between TRPV4 and SGK1 will ultimately prove to be an important component in many cellular functions.

SI113, a small molecular weight, membrane permeant compound, is an inhibitor of SGK1-mediated pathways [33]. SI113 is being studied as a potential chemotherapeutic agent in a variety of preclinical studies targeting hepatocarcinoma, glioblastoma multiform, ovarian and colon carcinoma cells [34–37]. These in vitro and in vivo studies have indicated a high specificity of SI113 for inhibiting SGK1 phosphorylation of artificial peptide substrates as well as known endogenous protein targets involved in oncogenic transformation including MDM2 (mouse double minute 2 homolog—an E3 ubiquitin-protein ligase) and NDRG1 (n-myc downstream regulated 1—a stress response protein) [33–37]. Importantly, in vitro assays have shown specificity for inhibition of SGK1 over AKT1 (RAC-alpha serine/threonine-protein kinase, or protein kinase B) which phosphorylates an identical target sequence in proteins as well as specificity over closely allied kinases such as Abl and Src [33, 34]. In vivo studies in mice have indicated no short-term toxicity as assessed by weight loss, diarrhea, dermatitis, ulceration or signs of liver failure [35].

The current studies confirm the specificity of SI113 in epithelial cells by showing that it inhibits aldosterone-sensitive sodium reabsorption in kidney collecting duct cells, a pathway known to be mediated by the SGK1 signaling axis [32, 41–43]. In a model of the human choroid plexus epithelium [25, 44], the HIBCPP line, SI113 inhibits both the TRPV4-mediated transepithelial ion flux and accompanying conductance changes. In a genetic rat model of postnatal hydrocephalus [45], treatment with SI113 dramatically halted the progression of ventriculomegaly in the affected animals via modulation of the activity of SGK1 in the choroid plexus of the affected animals. In summary, this project serves as a promising preclinical basis for the consideration of SI113 as a treatment modality for hydrocephalus due to multiple causes, and provides hope that a non-invasive, durable standard of care may be possible for patients with hydrocephalus.

## Methods

### Tissue culture

Mouse cortical collecting duct (mCCD_cl1_) cells were cultured in DMEM:F12 containing 4.5 g/L glucose, 22 mM bicarbonate, 4% fetal bovine serum, 100 U/L penicillin, 100 mg/L streptomycin, 1% insulin-transferrin-selenate 100X (Sigma #I3146), dexamethasone (50 nM), EGF (10 ng/mL), and triiodothyronine (1 nM). mCCD_cl1_ cells were grown to 100% confluency in 75 cm^2^ flasks, then passaged and seeded at 100% confluent density on polycarbonate permeable supports (Millicell #PIHP03050). Cultures were grown on filters in full media for 8 days, then fed in media without serum for 48 h, followed by 48 h in serum and hormone free media.

HIBCPP cells were grown in DMEM containing 4.5 g/L glucose, 44 mM bicarbonate, 10% fetal bovine serum, 100U/L penicillin, 100 mg/L streptomycin, and 5 μg/L insulin (Gibco, human recombinant). HIBCPP cells were grown to 90% confluency in 25 cm^2^ flasks, then passaged and seeded at 75% confluent density on Millicell permeable supports. Medium was replaced every second day for HIBCPP cells on filters for the first 7 days, and then was replaced daily for days 7–10. For days 10–16, cells were fed with serum-free DMEM media on the apical (top) surface and full media on the basolateral (bottom) surface to mimic in vivo conditions.

### Fluid production

HIBCPP cells were grown on Millicell permeable supports in 6 well plates. As per the manufacturer’s suggestion, the cells were grown with 1.5 ml of media on top (apical surface) and 2 ml in the bottom (basolateral side; facing the 6 well plate) to avoid any pressure differential across the cells. The cells cultured for 14–16 days to form confluent, high resistance monolayer cultures as previously described in Hulme et al. [25]. In the experimental protocol, apical and basolateral media were completely removed and replaced in the top and bottom chambers by use of an accurate Pipetman. Compounds or diluent (0.1 or 0.2% of total volume) were added to the apical compartment and 6 well culture plates were covered, rotated horizontally to mix and placed back in the incubator for 10 min. The apical media were completely removed and placed into pre-weighed collection tubes. The tubes containing media were then re-weighed and the fluid produced was calculated using the density of the media (0.9874 g/mL). The investigator weighing the tubes was blinded to the media treatment of each tube.

### Electrophysiology

mCCD_cl1_ cells were cultured on 30 mm polycarbonate filters for 12 days, and HIBCPP cells were cultured on the same inserts for 16 days. Filters were excised, mounted in Ussing chambers, assembled with salt-agar voltage and current electrodes and connected to a World Precision Instruments DVC-1000 Voltage/Current Clamp. Cells were bathed in temperature-controlled serum-free media and were oxygenated with a 5% CO_2_/O_2_ gas lift. Spontaneous transepithelial potential difference was measured and clamped to zero, resulting in a measurement of net transepithelial ion flux, short circuit current (I_SC_). A positive I_SC_ indicates anion secretion or cation absorption; a negative deflection indicates the opposite. A measure of barrier tightness, transepithelial electrical resistance (TEER), was calculated every 200 s through applying a 2-mV pulse and calculating resistance via Ohm’s law. Conductances were calculated as the inverse of TEER. The graphs shown in each panel represent a series of control and experimental cultures that were grown and analyzed in parallel (technical replicates). It is common practice in our laboratory to perform dose–response curves for each drug in each cell line and select the lowest dose that generates a maximal response. This is done to minimize off-target effects.

### In vivo study design

Wild-type, heterozygous, and homozygous (*Tmem67*^P394L^) pups were generated through the pairing of heterozygous adults. Genotyping of animals in this study was conducted using a previously described genotyping protocol [20]. Normal and hydrocephalic (*Tmem67*^P394L^) pups were randomly selected for either drug or vehicle treatment. Animals were excluded only if animal death occurred before the final MRI.

### Treatment protocol

Random selection separated the normal and hydrocephalic animals into groups to receive either drug or vehicle. MRIs were conducted on all pups on postnatal day 7 (pre-treatment) and again on postnatal day 15 (post-treatment). During the time interval between the MRIs, postnatal day 7–14, the pups were given a daily intraperitoneal injection of either SI113 (8 mg/kg BW) or an equivalent volume of vehicle (DMSO). Upon completion of the posttreatment MRI on postnatal day 15, the animals were sacrificed, and tissue was collected. The treatment protocol and tissue selection is shown schematically in Fig. 3A.

### Anatomical MRIs

As described in Hochstetler and Smith et al. [20], on P7 and P15, rat pups were briefly removed from their litter and anesthetized with 1–3% isoflurane (balance medical oxygen). High-resolution T2-weighted (T2W) MRI images were acquired using a 3 T Siemens Prisma clinical MRI scanner outfitted with a dedicated 4-channel rat head coil and bed system (RapidMR). Images were acquired using a SPACE3D sequence with the following acquisition parameters: (TA:,5.5 min; TR, 2080 ms; TE, 162 ms; ETL, 57; FS, On; Ave, 2; Excitation Flip Angle, 150; Norm Filter, On; Restore Magnetization, On; Slice Thickness, 0.2 mm: Matrix, 171 × 192; FOV, 35 × 35 mm) yielding 0.18 × 0.18 × 0.2 mm resolution images. Volumes of lateral ventricles were determined from intensity normalized and threshold-based image segmentation of native CSF contrast. Images were quantified for lateral ventricular volumes using Analyze 12.0 (Analyze Direct, Stillwell KS). In all cases, study personnel were blinded to genotype and treatment during acquisition and analysis.

### Reverse transcriptase PCR (RT-PCR)

Animals were euthanized via CO_2_ exposure and decapitation. Brains were dissected and lateral ventricle CPs were removed and flash frozen with liquid nitrogen. CP RNA was isolated using the Monarch Total RNA Miniprep Kit (New England Biolabs, T2010S). RNA concentration (μg/μl) and quality (A260/280 values > 2.0 and A260/230 values > 1.8) were measured using an ND2000 NanoDrop (Thermo Fisher Scientific). Total RNA was reverse transcribed into cDNA using the Monarch LunaScript RT SuperMix Kit (New England Biolabs, E3010L), along with corresponding negative RT (–RT) cDNA control and a no-RNA template control (NTC), according to the manufacturer’s directions. Rattus norvegicus exon mRNA sequences for each gene were obtained using Ensembl online database (ensembl.org), and primer pairs for each were designed using Primer3Plus (primer3plus.com; Table 1). Template cDNA was combined with the forward and reverse primers (IDT), as well as AccuStart II GelTrack PCR SuperMix (Quantabio, 95136). Reactions were run on a temperature gradient to determine optimum annealing temperature for each primer pair, and products were separated on a 2% TAE agarose gel with SYBR Safe DNA gel stain (Invitrogen, S33102). Flanking 100 bp and 50 bp ladders (New England Biolabs Inc., #B7025; #B7025) were used as molecular weight markers, and gels were imaged using a UV gel imager. RT-PCR reaction products for each target gene were purified with ExoSAP-IT Express (Applied Biosystems, 75001), sent for sequencing (Eton Biosciences), and the correct products were validated using NCBI BLAST (https://blast.ncbi.nlm.nih.gov) and confirmed for percent identity with the appropriate target.

**Table 1:** Primer Pairs utilized for RT-PCR and qRT-PCR

*Rattus norvegicus* gene	Protein	Primer Sequences		Product Size (bp)
		Forward Primer	Reverse Primer	
*Gapdh*	GAPDH	CCTGGAGAAACCTGCCAAGTAT	GACAACCTGGTCCTCAGTGTAG	103
*Rps18*	RPS18	CATGTGGTGTTGAGGAAAGCAG	TATTGTCGTGGGTTCTGCATGA	107
*Trpv4*	TRPV4	TCACCCTCACAGCCTACTATCA	GAGCCATCGACGAAGAGAGAAT	190
*Aqp1*	AQP1	GGCCAGCGAAATCAAGAAGAAG	CTCCAGTGGGTAATTGAAGCCT	118
*ATP1A1*	ATP1A1	ATTGCTGGTCTCTGTAACAGGG	ATGGAGAGCTGGTACTTGTTGG	206
*ATP1B2*	ATP1B2	AAGGAGTTCGTGTGGAATCCTC	AGGACGGCAGACATCATTCTTT	342
*Sgk1*	SGK1	CGGAGAGCTGTTCTACCATCTC	TAACCCAAGGCACTGGCTATTT	93

Primers included in this table were utilized for the experiments summarized in Fig. 4. Single band amplicon products were sequenced to verify correct gene amplification product. GAPDH = Glyceraldehyde 3-phosphate dehydrogenase; RPS18 = Ribosomal protein subunit 18; TRPV4 = Transient receptor potential vanilloid 4; AQP1 = Aquaporin 1; ATP1A1/B2 = ATPase Na + /K + Transporting Subunits Alpha 1/Beta 2; Serumglucocorticoid-induced kinase-1 = SGK1

### Quantitative, real-time PCR (qPCR)

CP RNA for each animal was collected and transcribed as described for RT-PCR. The cDNA was diluted with nuclease-free water (New England Biolabs). A standard curve of 1:10, 1:100, 1:1000, and 1:10000 dilutions of cDNA was used to determine the linear rage of the qPCR assay using the described primer pairs. All samples were run in triplicate. qPCR was performed with a LightCycler 480 Instrument II real-time PCR system (Roche LifeScience), using LightCycler 480 SYBR Green I Master Mix (Roche LifeScience, 04707516001). qPCR cycle conditions were 95 °C for 5 min, followed by 45 cycles of 95 °C for 10 s, 60 °C for 10 s, and 72 °C for 10 s. Data are displayed as a fold change in expression using the 2–ΔΔCT method, relative to the calibrator reference genes GAPDH and RPS18. Data are shown as fold change relative to the normalized control (untreated normal animals).

### Immunoblotting

Lateral ventricle samples of CP samples or cell lysate samples were collected on ice and lysed for 15 min in ice cold RIPA buffer (Millipore #20–188) + 1% HALT inhibitor cocktail (Invitrogen), manually homogenized, and clarified by centrifugation for 15 min at 25000 × *g*. 4 × Laemelli sample buffer (0.2 M Tris–HCl, 0.4 M dithiothreitol, 277 mM SDS, 6 mM bromophenol blue, 4.3 M glycerol) was added to the supernatant fraction of the samples and the samples were denatured at 70 °C for 10 min. Samples were loaded onto 4–15% Bio-Rad Gradient TGX Stain-Free gels and separated. After separation, proteins were transferred to nitrocellulose membranes under semi-dry conditions using the Bio-Rad Trans-Blotter system at 1.0A for 30 min. Membranes were stained with total protein stain (Ponceau-S), imaged and then incubated in blocking buffer (5% milk in Tris buffered saline). Membranes were incubated with primary antibody (Table 2) diluted in blocking buffer overnight at 4 °C. Membranes were washed with blocking buffer for 30 min at room temperature and then incubated in secondary antibody (Jackson ImmunoLabs donkey anti-rabbit AlexaFluor 790) diluted in blocking buffer for one hour at room temperature. Membranes were exposed on a LICOR Odyssey imaging station to visualize antibody-bound fluorescent signal. Band intensity analysis was completed in LICOR Odyssey software. Blots shown are representative of technical duplicates and biological replicates per genotype/treatment condition as indicated in the figure legends. Band intensities were normalized to Ponceau for total protein loading as measured by ImageJ pixel intensity quantification per lane. For p-SGK1 and SGK1, bands were normalized to total protein loading and then represented as a ratio of p-SGK1 to total SGK1.

**Table 2:** Antibodies used for western blotting

Target	Antibody	Species	Concentration	Target sequence	Verification method
TRPV4	Invitrogen PA5-77319	Rabbit	1:2000	CDGHQQGYAPKWRAEDAPL (Rat)	KO tissue, IP/IB with control IgG
SGK1	Upstate 07–315	Rabbit	1:500	C-GKSPDSVLVTASVK (Human)	IP/IB w/control IgG
p-SGK1	Abcam ab55281	Rabbit	1:200	Ser422 (Human)	control antigen
	Upstate 36–002	Rabbit	1:500	Ser255/Thr256 (Human)	PDK activated SGK1 samples
p-NEDD4-2	Abcam Ab168349	Rabbit	1:500	Ser448 (Human)	293 T cell lysate
p-NDRG1	Cell Signaling 5482	Rabbit	1:2000	Thr346 (Mouse)	A204 cell lysate

Antibodies included in this table were utilized in the experiments shown in Figs. 5 WB = western blot; KO = knockout; IP/IB = immunoprecipitation followed by western blot; TRPV4 = Transient receptor potential vanilloid 4; Serumglucocorticoid-induced kinase-1 = SGK1

### Statistical analysis

Data were graphed and analyzed in GraphPad Prism 8.0. For electrophysiology tracings, repeated measures analysis of variance (RM-ANOVA) was utilized to determine the difference between two traces over time, coupled with multiple t-tests row analysis. Greenhouse–Geisser epsilon correction was used for non-spherical data. For data represented in bar charts, group means were compared utilizing either ANOVA or t-test, depending on the complexity of the data. For non-parametric t-tests, Wilcoxon–Mann–Whitney post hoc analysis was used. For non-parametric ANOVA, Kruskall-Wallis test was used. For all analyses, p < 0.05 was considered significant.

### Animal approval

The hydrocephalic model used in these studies was the *Tmem67*^P394L^ rat [20, 45–47]. The animal experiments were completed utilizing protocols approved by the Institutional Animal Care and Use Committee of Indiana University Purdue University Indianapolis.

## Results

### SI113 inhibits a well characterized SGK1-mediated signaling pathway

**Fig. 1:**
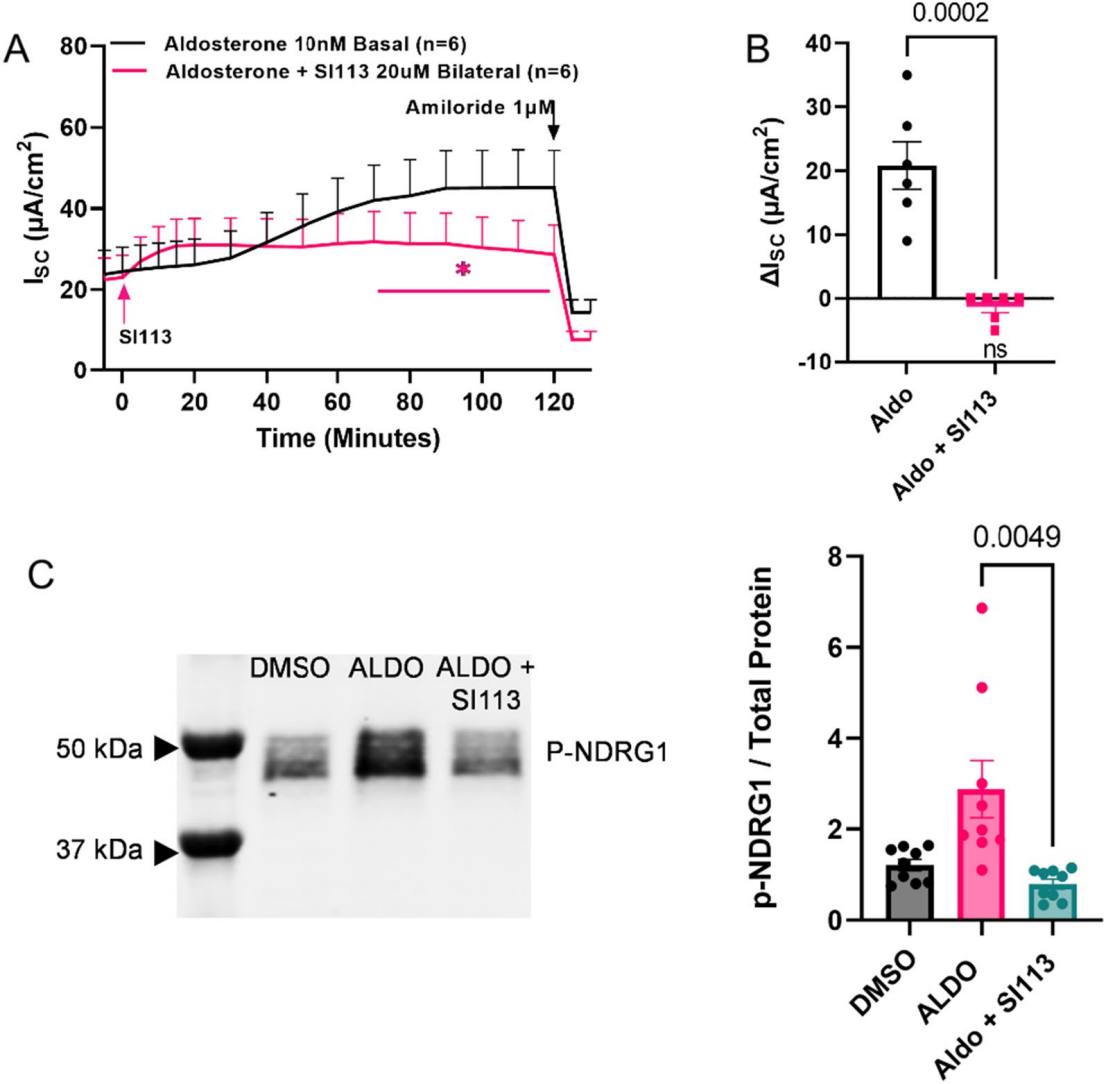
SI113 blocks the aldosterone-stimulated increase in ENaC-mediated sodium absorption and decreases SGK1 activity in a murine cortical collecting duct (mCCD_cl1_) cell line. The tracings in **A** illustrate the average short circuit current, I_SC_, a measure of net transepithelial absorptive sodium flux across confluent monolayers of mCCD_cl1_ cells grown on permeable supports, for a number of replicates, n, indicated on the graph. Cultures were mounted in Ussing chambers and the spontaneous potential difference across the monolayer was measured and clamped to zero. The resulting I_SC_. was measured as a function of time. At time t = − 10 min all cultures were incubated with 10 nM aldosterone delivered to the basal side. At time t = 0 min, experimental cultures were incubated with 20 µM SI113 bilaterally. At time t = 120 min, 1 µM amiloride, a specific inhibitor of the epithelial sodium channel, ENaC, was added to indicate the amount of I_SC_ that was due to net sodium flux. **B** represents change from baseline for I_SC_ of control and experimental cultures. **C** shows a representative immunoblot (n = 3 biological replicates, technical triplicates) for p-NDRG1 in vehicle treated (DMSO), aldosterone treated (2 h incubation), or SI113 + aldosterone treated cell lysates. Bar charts show relative abundance of p-NDRG1 to total protein in cell lysates. Data represented as mean ± sem in all graphs. I_SC_ = short circuited current; n = number of replicates; Aldo = aldosterone; SI = SI113; p-NDRG1 = N-Myc Downstream Regulated 1

To test the specificity of SI113 against SGK1, we employed a mouse cortical collecting duct cell line, mCCD_cl1_. The role of SGK1 in the intracellular pathways controlling ENaC activity in the aldosterone-sensitive segments of the nephron is well characterized [32, 41–43]. Using electrophysiological techniques to measure electrogenic transepithelial ion flux, we demonstrated that the mCCD_cl1_ cells are indeed aldosterone sensitive, demonstrating an increase in I_SC_ over the basal level upon addition of 10 nM aldosterone to the basolateral side, comparable to what we and other groups have previously reported in these cells (Fig. 1A, black trace; Fig. 1B, black circles). After 2 h, low dose amiloride was added to the cultures to eliminate the epithelial sodium channel (ENaC)-mediated sodium flux. Since the majority of the basal and aldosterone-induced current was amiloride sensitive, we can conclude the electrogenic flux measured in our experiments corresponded with ENaC-mediated sodium absorption. Addition of SI113 (20 uM bilaterally) before aldosterone (Fig. 1A, pink trace) inhibited the aldosterone-invoked increase in sodium transport. These data are compelling that the SI113 inhibits SGK1 activity. As an additional measure of SGK1 activity, we evaluated the abundance of known downstream target of SGK1, p-NDRG1. Previous studies in these same cells showed that a commercial SGK1 inhibitor reduces the p-NDRG1 abundance [42]. In our cells, we also see an increase in p-NDRG1 abundance in aldosterone treated cell lysates (Fig. 1C, pink circles), consistent with an increase in SGK1 activity. This is attenuated by treatment with SI113 as shown by the reduction in p-NDRG1 abundance (Fig. 1C, teal circles). From these experiments, coupled with the literature on its specificity [33–37], we concluded that the SI113 is a specific inhibitor of SGK1 kinase activity.

### SI113 inhibits the SGK1-TRPV4 signaling axis in human choroid plexus cells

**Fig. 2:**
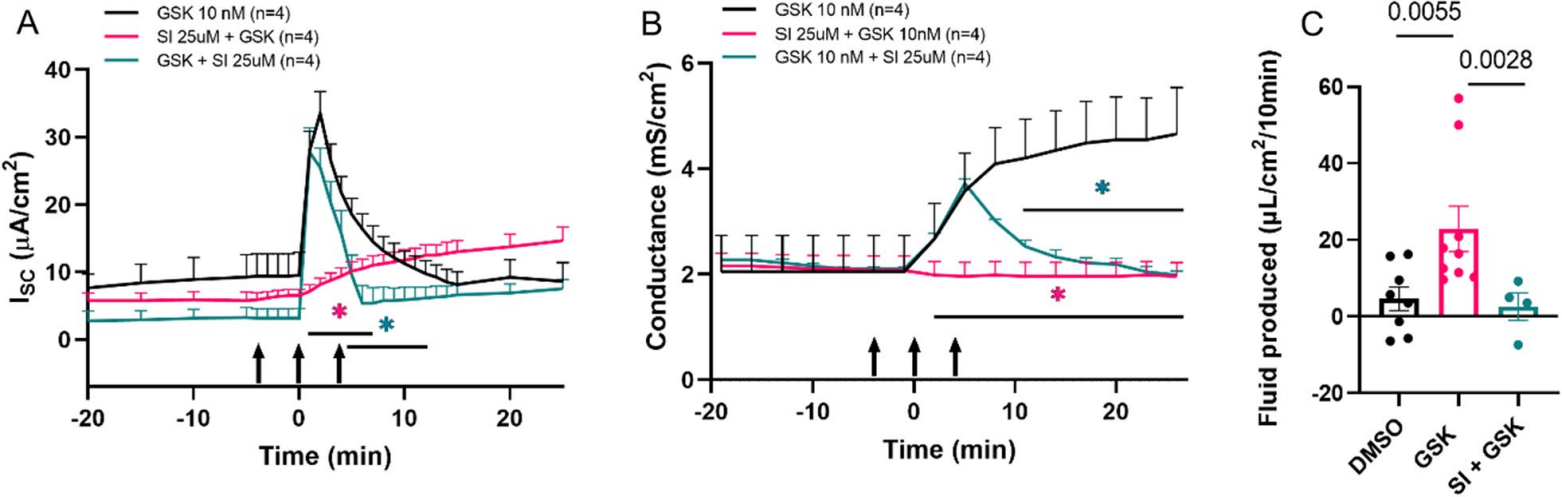
SI113 blocks TRPV4-mediated transepithelial ion flux and conductance changes in a human choroid plexus cell line (HIBCPP). Short circuit current, I_SC_, is a measure of net transepithelial electrogenic ion flux; conductance, the inverse of the transepithelial electrical resistance, is a measurement of the transepithelial permeability. HIBCPP confluent monolayers were mounted in Ussing chambers and allowed to reach stable baselines. At time t = − 5 min, experimental cultures were incubated with 25 µM SI113 bilaterally and at time t = 0 min, all cultures received 10 nM GSK apically to stimulate transepithelial ion transport. Pretreatment with SI 113 (pink trace) substantially inhibited TRPV4-mediated changes in transepithelial ion flux **A** and completely inhibited the TRPV4-mediated increase in conductance **B** (black trace = control). Post-treatment with SI 113 added at time t = 5 min (teal trace), reversed the ongoing TRPV4-mediated transport and conductance. **C** Fluid produced in microliters per square centimeter by cultures treated with DMSO (control), GSK (15 nM) or SI + GSK (25 μM SI113) for 15 min. n = 5 DMSO and n = 7 GSK data points replicated from Hulme et al. [22]. Data represented as mean ± sem in all graphs. n = number of replicates; ns = not significant; GSK = GSK1016790A, TRPV4 agonist. HIBCPP = human choroid plexus papilloma cell line

To characterize the effect of SGK1 in choroid plexus epithelial cells, we utilized a human CPe continuous cell line that has been validated and characterized in our laboratory as a moderate-resistance line that has correct polarization of key transport proteins and maintains several characteristics of the native tissue [25, 44]. In this cell line TRPV4 stimulates a polymodal increase in transepithelial electrolyte flux as well as a substantial increase in transepithelial permeability [25]. In other tissues, SGK1 has been shown to phosphorylate and activate TRPV4 channels at the membrane, thus acting as an upstream regulatory kinase for TRPV4 [39, 40]. In the human choroid plexus epithelial cell line, we found that SI113 pretreatment had no effect on basal transepithelial ion flux but blocked the normal TRPV4-stimulated multiphasic ion flux and conductance increases (Fig. 2A, B, pink traces). Interestingly, addition of SI113 5 min after stimulation with a TRPV4 agonist also interrupted the in-progress response and returned the cells to baseline ion transport and conductance (Fig. 2A, B, teal traces). This post-treatment response is similar to the reversal of the TRPV4 response by TRPV4 antagonists [25]. Thus, this experiment indicates that SGK1 and TRPV4 are functionally linked in the choroid plexus epithelial cells and that SGK1 is an important regulatory kinase in the choroid plexus epithelial cells. We also have previously established that TRPV4 activation in these cells causes an increase in fluid production [25], and here, pretreatment with SI113 inhibits the TRPV4 agonist-induced fluid production (Fig. 2C).

### SI113 ameliorates ventriculomegaly in a genetic rat model of hydrocephalus

To study the effect of SI113 on hydrocephalus, we used a genetic rat model *Tmem67*^P394L^; (also called Wpk for “with polycystic kidney disease”). This model is orthologous to a human genetic disease called Meckel-Gruber Syndrome type 3 [45–47]. The affected animals carry a single C to T substitution in *Tmem67,* exon 12 that converts a proline to a leucine in a protein product (P394L) which encodes one of a complex of proteins involved in formation of the primary cilium. The homozygous affected animals have severe hydrocephalus and renal cystic disease and typically survive for 18–21 days after birth. Importantly, we have shown treatment with two structurally distinct TRPV4 antagonists inhibits the progression of hydrocephalus in this model [20].

**Fig. 3:**
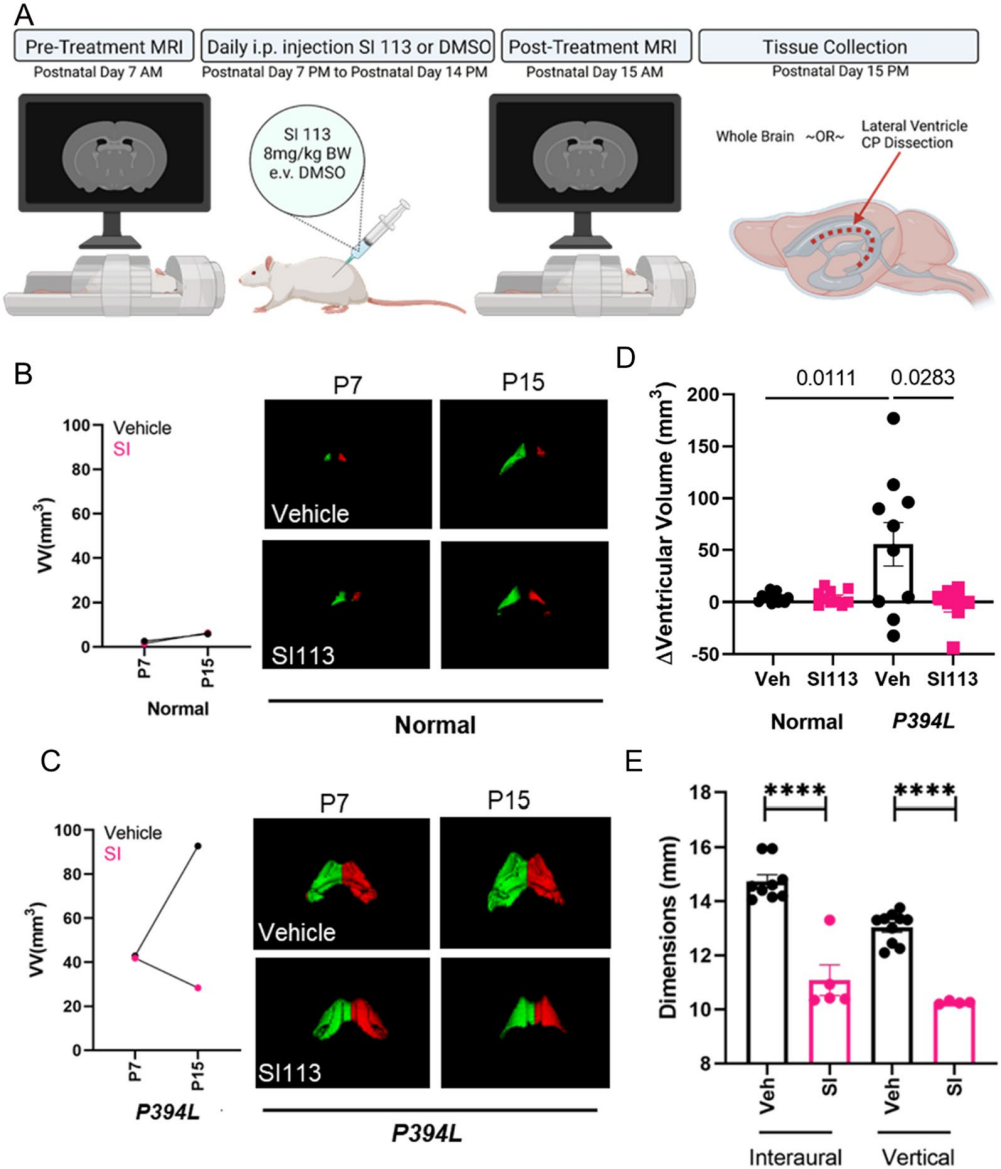
SI113 treatment ameliorates hydrocephalus in a genetic rat model (*Tmem67*^*P394L*^). Normal and hydrocephalic (*Tmem67*.^P394L^) littermates were treated with either vehicle (DMSO) or SI113 (8 mg/kg BW) daily from P7 to P15 via intraperitoneal injection as described in the treatment schematic in **A**. Graphs **B** and **C** represent matched normal and hydrocephalic animals, respectively. The two graphs show representative animals with the similar starting ventricular volumes at P7, comparing a mean effect of the vehicle or drug treatment on ventricular volume at P15. Coronal slices from MRI scans taken from the same animals depicted in the graphs are shown beside each graph. Graph **D** summarizes the change in ventricular volume (VV) from P7 to P15 for all animals in the cohort, indicating the drug treatment was effective at halting the progression of ventriculomegaly in the affected animals, and had no effect on the normal animals’ ventricular volumes. **E** shows the effect of SI113 treatment on cranial dimensions in the hydrocephalic animals. Data represented as mean ± sem in graphs **D** and **E**. Veh = vehicle, DMSO; SI = SI113; VV = ventricular volume; ns = not significant; numbers on the bars in D show the p values for the comparisons listed; **** = p < 0.0001

Since SGK1 and TRPV4 appear to be functionally linked in the choroid plexus epithelium, we sought to test whether SI113 would also effectively inhibit ventricular enlargement in the genetic rat model. The treatment scheme is graphically represented in Fig. 3A. Pre- and post-treatment MRI scans were used to elucidate progression of the phenotype. Figures 3B/C are representative examples of normal (B) or hydrocephalic (C) animals with matched P7 scan volumes and P15 scan volumes per treatment as a method of demonstrating ventricular growth and the effect of drug or vehicle. Figure 3D represents the summary data, wherein each point represents a single animal’s change in ventricular volume (ΔVV) from P7 to P15. Despite some phenotypic variability, the majority of the hydrocephalic vehicle-treated animals have an increase in ventricular volume from P7 to P15, and this increase is significantly attenuated by treatment with the SGK1 inhibitor, SI113. Alternatively, SI113 caused no change to ventricular volume in the normal animals. In addition to ventriculomegaly, the rats also develop noticeable macrocephaly compared to their normal littermates [20]. The same hydrocephalic animals used for the MRI study were measured for cranial dimensions at P15 and these values are reported in Fig. 3E. In both interaural and vertical head dimensions, treatment with SI113 reduced the cranial dimensions of the hydrocephalic animals. There was no change to head dimensions of the wild-type animals due to drug treatment, similar to our findings in a previous TRPV4 antagonist treatment study. Furthermore, although the hydrocephalic animals generally weigh less than their normal littermates, the drug treatment did not significantly alter body weight compared to vehicle treated (data not shown).

### SI113 treatment does not modify transcriptional levels of Trpv4 or Sgk1

**Fig. 4:**
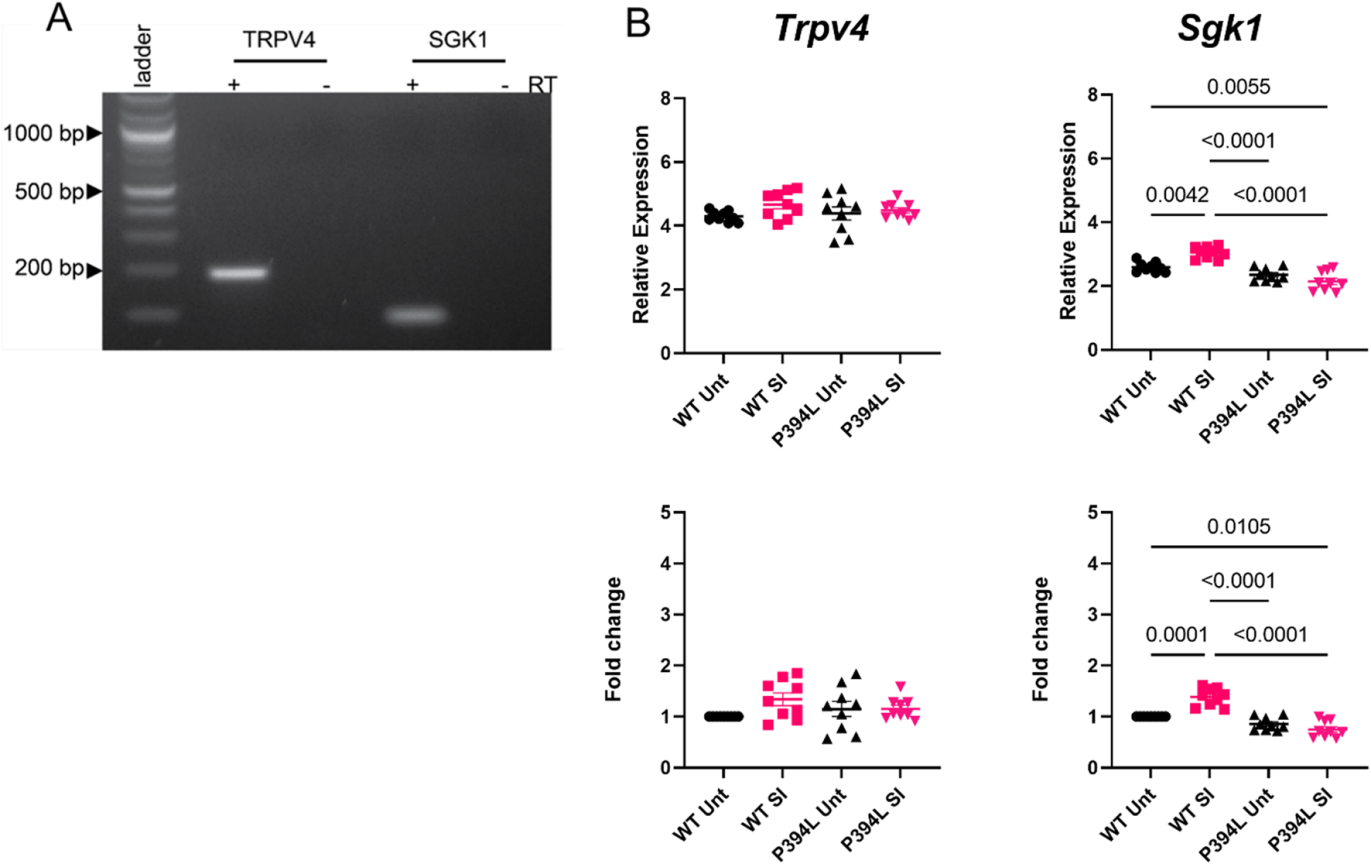
*Trpv4* and *Sgk1*mRNA expression in rat choroid plexus. (**A**) Agarose gel of RT-PCR products showing the presence of *Sgk1* and *Trpv4* in rat choroid plexus. **B** qRT-PCR analysis of normal and mutant (P394L) choroid plexus lysates with and without SI113 treatment (n = 3 biological replicates, technical triplicates) with *Trpv4* and *Sgk1,* primers. Although there were statistically significant differences, no genes demonstrated more than two-fold changes in either direction from the normal, untreated animal. Data represented as mean ± sem in all graphs. TRPV4 = transient receptor potential vanilloid 4; Serum/glucocorticoid regulated kinase-1 = SGK1. Primer information can be found in Table 1

Using the lateral ventricle choroid plexus lysate from animals in the treatment cohort (Fig. 3), we first confirmed that *Sgk1* and *Trpv4* were expressed using RT-PCR (visualized in Fig. 4A). Next, quantitative PCR was used to determine potential changes in the gene expression of *Trpv4* and *Sgk1* that may be modified by hydrocephalus or SI113 treatment (Fig. 4B, C). Interestingly, there were no changes that met the significance threshold of a twofold change in either direction from the normal, untreated condition for either of the genes measured.

### SI113 treatment decreases phosphorylation of SGK1 and downstream targets, with no change in abundance of the TRPV4 or SGK1 proteins

**Fig. 5:**
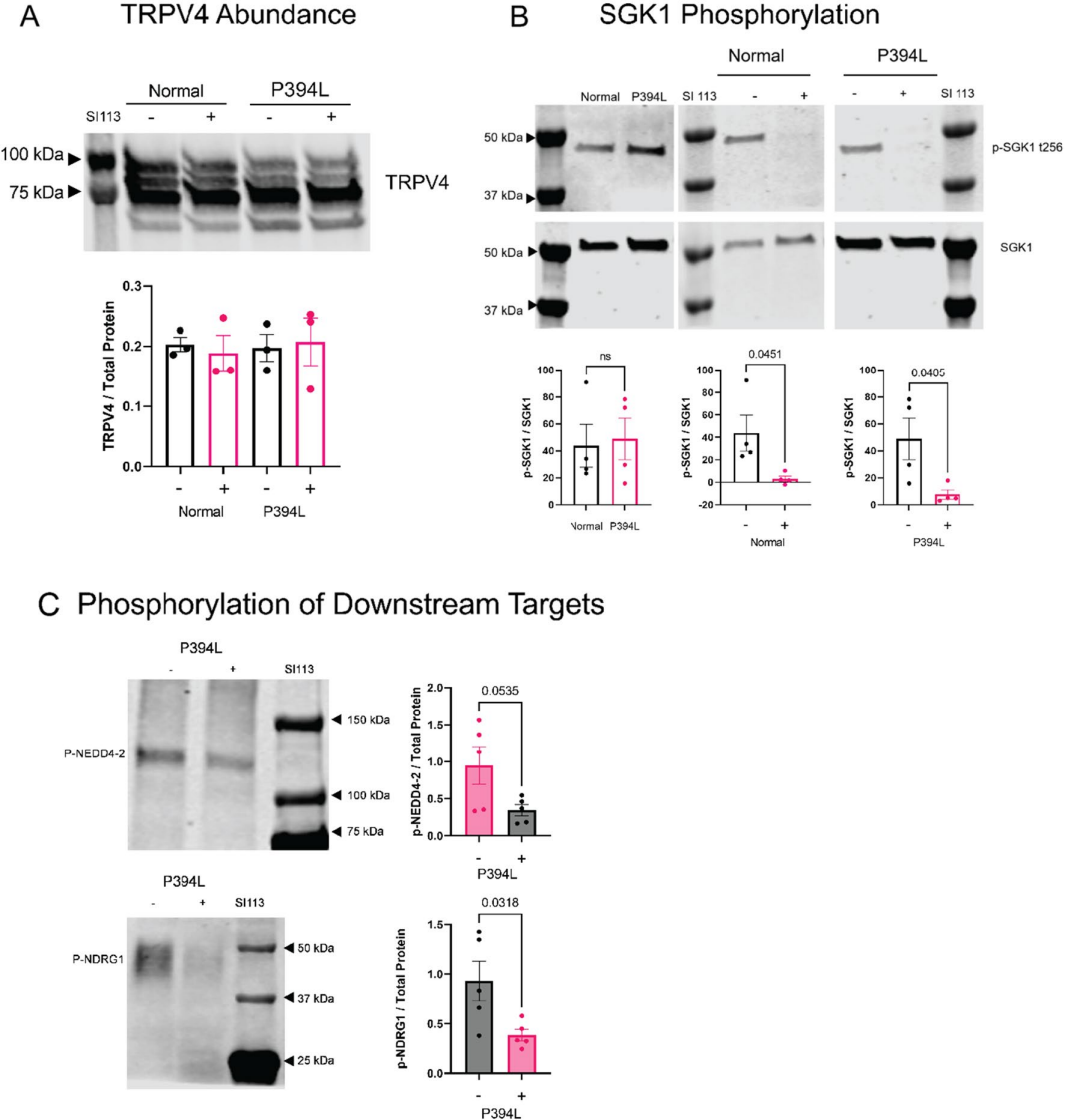
SI113-mediated changes in abundance of TRPV4, p-SGK1, and downstream targets of SGK1 in the choroid plexus of hydrocephalic (P394L) rats. **A** Representative immunoblots for lateral ventricle choroid plexus lysates probed for TRPV4 in normal and hydrocephalic animals, untreated or treated with SI113. Band intensities were normalized to Ponceau-S and the graphs represent the ratio of TRPV4 to total protein. Lysates were blotted in technical duplicates and n = 3 biological replicates. **B** Representative immunoblots for SGK1 and p-SGK1 (t256) in SI113 treated or untreated tissue from normal and hydrocephalic animals. Band intensities were normalized to Ponceau-S for total protein loading per lane, and the graphs represent the ratio of p-SGK1 to total SGK1 in the samples. Lysates were blotted in technical duplicates and n = 4 biological replicates. **C** Representative immunoblots for downstream targets of SGK1, p-NEDD4-2 and p-NDRG1 in lateral ventricle choroid plexus lysates in normal and hydrocephalic animals, untreated or treated with SI113. Band intensities were normalized to Ponceau-S and the graphs represent the ratio of the protein of interest to total protein. Lysates were blotted in technical duplicates and n = 2 biological replicates. Data represented as mean ± sem in all graphs. Significance values are displayed on the graphs and were determined by student’s unpaired t-test in GraphPad Prism 8.0

We postulated that despite the lack of significant changes in gene expression, perhaps the abundance and/or phosphorylation of SGK1 and TRPV4, as well as some downstream targets of SGK1, would be changed due to hydrocephalus and/or drug treatment. To evaluate this, we performed immunoblotting on lateral ventricle choroid plexus lysates to determine abundance of TRPV4, SGK1, p-SGK1 (t256), p-NEDD4-2 (s448), and p-NDRG1 (t356). We found that total TRPV4 abundance did not change with regards to the previously identified CPe isoforms and glycosylation products in response to hydrocephalus or drug treatment (Fig. 5A), consistent with the findings in our previous TRPV4 antagonist treatment study [20]. Additionally, the ratio of p-SGK1 (t256) to SGK1 (a representation of SGK1 activity), also did not change due to hydrocephalus (Fig. 5B). However, the treatment with SI113 did significantly decrease phosphorylation of SGK1 in both normal and hydrocephalic animals (Fig. 5B); in some cases this was to undetectable levels. Phosphorylation of downstream SGK1 targets p-NDRG1 and p-NEDD4-2 were similarly reduced in hydrocephalic animals treated with SI113 (Fig. 5C), further indicating a role for SI113 as an inhibitor of SGK1.

## Discussion

The data presented in this manuscript provide innovative preclinical proof-of-concept and proof-of-mechanism studies for SGK1 as a therapeutic target in the treatment of hydrocephalus. SI113 has not been extensively studied in epithelial cells, therefore requiring initial specificity studies for the SGK1 inhibitor. We first showed that SI113 is an inhibitor of SGK1 activity using a well-established signaling axis of aldosterone-mediated sodium reabsorption in a renal distal nephron epithelial cell line (Fig. 1). In this set of experiments, we showed that SI113 treatment inhibits aldosterone-mediated sodium transport via ENaC and that this is due to a decrease in SGK1 activity as measured by a decrease in phosphorylation of a well validated downstream target, NDRG1. Having established specificity and effectiveness in a well characterized epithelial model, we showed that SI113 reduces TRPV4 stimulated increases in transepithelial ion flux, barrier conductance and fluid flux in a model of human choroid plexus epithelial cells, the HIBCPP cell line (Fig. 2; 25) thus linking SGK1 activity to TRPV4 in the choroid plexus epithelium.

We evaluated the efficacy of SI113 as a treatment for hydrocephalus using our previously established genetic rat model [20, 45]. In Fig. 3, we show that SI113 blocks the development of ventriculomegaly and macrocephaly in the hydrocephalic animals, thus establishing preclinical proof-of-concept. This could be due to a variety of reasons including either decreased production and/or increased absorption of CSF, and more elegant in vivo measurements to determine drug effect on CSF production using an infusion test are outside the scope of this current manuscript. Although we did not formally measure health parameters, our observations of no overt drug toxicity are consistent with previous, more detailed studies indicating no toxic effects of SI113 at a similar dose and method of administration in control and tumor-induced mice [35]. The results are also consistent with the finding that under normal conditions SGK1 knock out animals are healthy and breed normally [48]. Thus, these data suggest that like TRPV4 activation, SGK1 is a kinase that plays a role in hydrocephalic development.

Proof-of-mechanism for SGK1 inhibition was evaluated using tissue collected from the treated animal cohort by assessing gene expression and protein abundance and phosphorylation. In Fig. 4 we demonstrate that neither hydrocephalus nor SI113 treatment modify transcriptional levels of *Sgk1* or *Trpv4*. We established that neither hydrocephalus nor SI113 treatment modify abundance of the TRPV4 protein in the choroid plexus (Fig. 5), similar to our previous study in which TRPV4 antagonists were shown to ameliorate hydrocephalic development [20]. However, we do show that SI113 treatment reduces the levels of p-SGK1 and its downstream target, p-NDRG1, in the hydrocephalic animals. These data inform two major conclusions: 1) the activity of SGK1 in the choroid plexus can be inhibited by SI113, and 2) SGK1 activity in the choroid plexus involves many downstream targets, including p-NDRG1 and potentially TRPV4. The interactions between SGK1 and p-NDRG1 in the choroid plexus are more obvious because of the decrease in phosphorylated protein with treatment. This is a novel finding because it establishes precedent that SGK1 is an important regulatory kinase in the choroid plexus and indicates that it plays a role in both ubiquitination pathways and stress response pathways in this tissue. Although our studies did not allow us to show a direct effect on phosphorylated TRPV4 due to a lack of phospho-specific antibodies, the previous functional assay utilizing Ussing Chamber electrophysiology shows that SGK1 and TRPV4 are functionally linked in the choroid plexus.

Limitations of these studies include the caveat that short-circuit current in our electrophysiological experiments detects net transepithelial current flux. Therefore, movement through electroneutral transporters like NKCC cannot be directly assessed and their effects are only measurable as secondary effects on electrogenic transport. Also, because the technique measures net charge movement, in the case of the simultaneous electrogenic movement of anions and cations, the short-circuit current does not mirror the total electrolyte movement. Although the human choroid plexus cell line is relatively well characterized, cell lines are not subjected to the same environment as the corresponding cells in vivo such as osmotic variations, pressure changes, hormone and cytokine exposure arising from other cell types.

Taken together, the results suggest that SI113 treatment modifies TRPV4 activity at the membrane, rather than total gene or protein expression. Our pivotal study on the interaction between SGK1 and TRPV4 in the choroid plexus and their combined roles in CSF secretion represents a foundation of preclinical evidence to support further exploration of SGK1 inhibitors for the treatment of CNS volume disorders.

## Conclusions

**Fig. 6:**
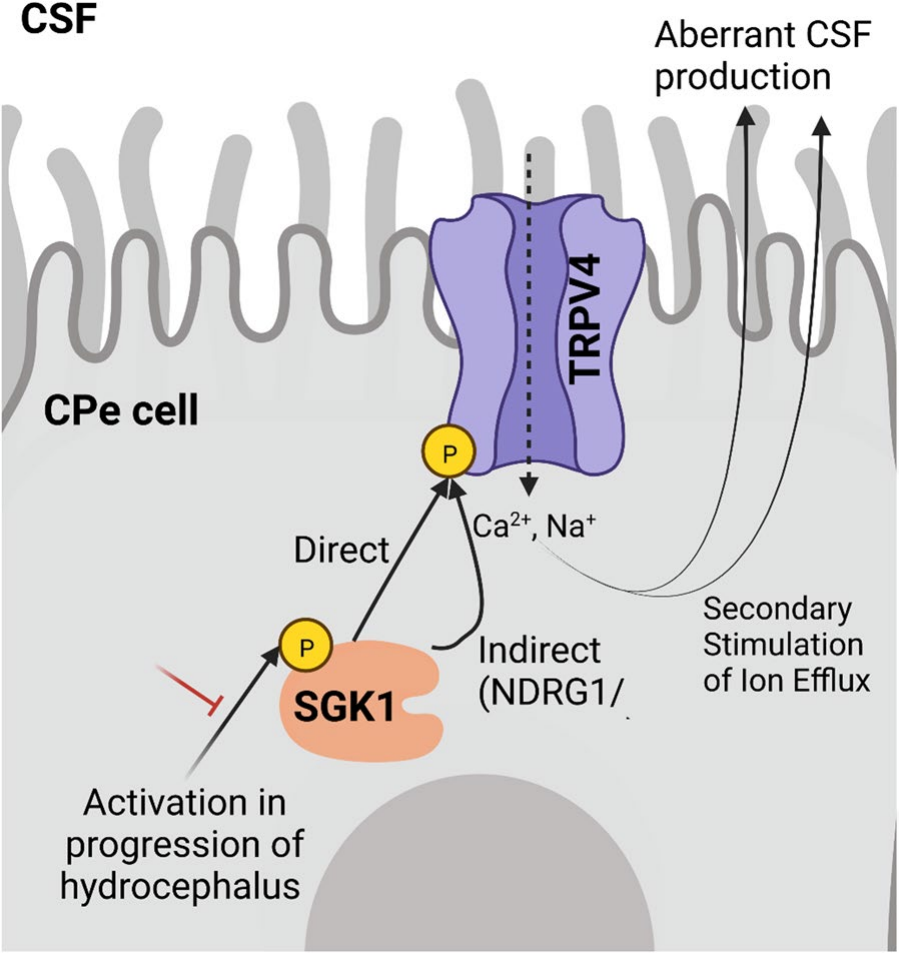
Graphical representation of the intracellular Signaling Pathway Created in Biorender

We have shown that SGK1 activity is important for TRPV4 activation during choroid plexus-mediated stimulation of electrolyte and fluid secretion, making this pathway an attractive target in the treatment of homeostatic disorders such as hydrocephalus. Indeed, recent data confirm that SGK1 is the fourth most highly expressed kinase in the choroid plexus, indicating that its role in CSF production is likely important [49]. We show compelling evidence that SI113 inhibits SGK1 activity and corresponding TRPV4 activation in the choroid plexus, and that SI113 treatment inhibits the progression of hydrocephalus in a preclinical rodent model (Fig. 6).

## Data Availability

The datasets used and/or analyzed during the current study are available from the corresponding author upon reasonable request.

## Abbreviations

CSF: Cerebrospinal fluid
ΔVV: Change in ventricular volume
CP: Choroid plexus
CPe: Choroid plexus epithelial cells
ENaC: Epithelial sodium channel
HIBCPP: Human choroid plexus papilloma cell line
mCCD_c11_: Mouse cortical collecting duct cell line, clone 1
MDM2: Mouse double minute 2 homolog-An E3 ubiquitin-protein ligase
NDRG1: N-myc downstream regulated 1
PBS: Phosphate buffered saline
PCP-R: Porcine choroid plexus cell line, Riems
qPCR: Quantitative, real-time PCR
Akt1: RAC-alpha serine/threonine-protein kinase, or protein kinase B
RM-ANOVA: Repeated measures analysis of variance
SGK1: Serum-, glucocorticoid-induced kinase 1
I_SC_: Short circuit current
TEER: Transepithelial electrical resistance
TRPV4: Transient receptor potential vanilloid 4
